# Tryptophan metabolism as bridge between gut microbiota and brain in chronic social defeat stress-induced depression mice

**DOI:** 10.3389/fcimb.2023.1121445

**Published:** 2023-02-24

**Authors:** Jing Xie, Wen-tao Wu, Jian-jun Chen, Qi Zhong, Dandong Wu, Lingchuan Niu, Sanrong Wang, Yan Zeng, Ying Wang

**Affiliations:** ^1^ Chongqing Emergency Medical Center, Central Hospital of Chongqing University, Chongqing, China; ^2^ Institute of Life Sciences, Chongqing Medical University, Chongqing, China; ^3^ Department of Rehabilitation, The First Affiliated Hospital of Chongqing Medical University, Chongqing, China; ^4^ Department of Rehabilitation, The Second Affiliated Hospital of Chongqing Medical University, Chongqing, China; ^5^ Department of Psychology, The Second Affiliated Hospital of Chongqing Medical University, Chongqing, China

**Keywords:** depression, gut microbiota, tryptophan, Firmicutes, *Lactobacillus*

## Abstract

**Backgrounds:**

Gut microbiota plays a critical role in the onset and development of depression, but the underlying molecular mechanisms are unclear. This study was conducted to explore the relationships between gut microbiota and host’s metabolism in depression.

**Methods:**

Chronic social defeat stress (CSDS) model of depression was established using C57BL/6 male mice. Fecal samples were collected from CSDS group and control group to measure gut microbiota and microbial metabolites. Meanwhile, tryptophan metabolism-related metabolites in hippocampus were also analyzed.

**Results:**

CSDS successfully induced depressive-like behaviors in CSDS group. The 24 differential bacterial taxa between the two groups were identified, and 14 (60.87%) differential bacterial taxa belonged to phylum Firmicutes. Functional analysis showed that tryptophan metabolism was significantly affected in CSDS mice. Meanwhile, 120 differential microbial metabolites were identified, and two key tryptophan metabolism-related metabolites (tryptophan and 5-hydroxytryptophan (5-HTP)) were significantly decreased in feces of CSDS mice. The correlation analysis found the significant relationships between tryptophan and differential bacterial taxa under Firmicutes, especially genus *Lactobacillus* (r=0.801, p=0.0002). In addition, the significantly decreased 5-hydroxytryptamine (5-HT) in hippocampus of depressed mice was also observed.

**Conclusions:**

Our results showed that tryptophan metabolism might have an important role in the crosstalk between gut microbioa and brain in depression, and phylum Firmicutes, especially genus *Lactobacillus*, might be involved in the onset of depression through regulating tryptophan metabolism.

## Introduction

Depression is a common mental disorder that severely affects quality of life and psychosocial functioning of patients (Affatato et al., 2021). During a depressive episode, people will experience different symptoms, such as empty, low self-worth, and a loss of pleasure, even thoughts about suicide (Grover and Adarsh, 2022; Ning et al., 2022). According to the reports of World Health Organization, depression affects about 3.8% of the population and causes huge economic burdens on individual, family and society. However, the pathogenesis of depression is yet unclear (He et al., 2022; Zhong et al., 2022), which results in two serious problems: i) only about 70% patients response to the first-line antidepressants; and ii) no objective laboratory methods are developed to diagnose depression (Cai et al., 2022; Mojtabavi et al., 2022; Tian et al., 2023). Therefore, it is urgently needed to further explore the pathogenesis of depression.

Gut microbiota recently has been considered to be participated in the onset and development of depression (Jiang et al., 2021; Liu et al., 2021; Pu et al., 2022; Song et al., 2022). Previous study reported that gut microbiota might play a key role in the physiopathology of depression by regulating brain neurotransmitters (Huang and Wu, 2021). Carlessi et al. suggested that the disordered gut microbiota would cause a systemic inflammation, which eventually influenced responses to depression treatment (Carlessi et al., 2021). In our previous studies, we found that there were significant differences on gut microbiota compositions between healthy controls and depression patients, and gut microbiota might participate in the onset of depressive-like behaviors by affecting host’s metabolism (Zheng et al., 2016; Chen et al., 2020; Bai et al., 2021). Using depression mice model, we found that gut microbiota might influence the levels of neurotransmitters in hypothalamus through its metabolic products (Wu et al., 2020).

Although much work has been done, the detailed mechanism of gut microbitoa in depression has still not been completely understood. Thus, in this study, we established depression mice model using chronic social defeat stress (CSDS) method to further investigate the role of gut microbiota in the pathogenesis of depression. The 16S rRNA gene sequencing analysis was used to identify the differential gut microbiota, and then the functions of these differerntial bacterial taxa were predicted. Meanwhile, microbial metabolites in feces were also detected using liquid chromatography-mass spectrometry (LC-MS). Considering the important role tryptophan metabolism in the pathogenesis of depression (Chojnacki et al., 2022; Lu et al., 2022; Pu et al., 2022; Xiao et al., 2022), tryptophan metabolism-related metabolites in hippocampus were also analyzed. Integrating these data, we sought to find out the potential pathways in the crosstalk of gut and brain in depressed mice.

## Materials and methods

### Depression model

This study was performed according to the National Institutes of Health’s Animal Research Guide, and Ethics Committee of Chongqing Medical University reviewed and approved this study. C57BL/6 male mice were provided by Laboratory Animal Center of Chongqing Medical University (Chongqing, China) and housed in groups of five. CD1 male breeders were used as aggressors for this study and singly housed. After one week adaptation, we randomly assigned the C57BL/6 male mice into control group and CSDS group. Sucrose preference and body weight (BW) were matched in the two groups. In the next 10 days, mice in CSDS group were subjected to social defeated stress from CD1, and mice in control group were not disturbed (Wang et al., 2016). The mice after the CSDS models were prepared were single-housed. Subsequently, mice in two groups underwent behavioral tests to assess whether the depression model was successfully built or not.

### Behavioral tests

The mice were subjected to behavioral tests one day after CSDS. The following behavioral tests were successively conducted here: social interaction test (SIT), sucrose preference test, open field test (OFT), and forced swim test (FST). The behavioral tests were carried out in another same room to rule out the potential effects CD1 male. The procedures of these tests were the same as those in our previous studies (Gong et al., 2021; Xie et al., 2022) (**Supplementary File 1**). During these tests, the operators were blinded to the group allocation of mice. Each behavioral test continued for six minutes, and we recorded the activities of each mouse in the last five minutes. Three indicators (center area time (CT) (%) in OFT, sucrose preference (SPT) in sucrose preference test and immobility time (IT) in FST) were calculated to assess whether the mice in CSDS group showed depressive-like behaviors or not. Meanwhile, BW of each mouse in the two groups was collected.

### Data collection

After completing these behavioral tests, the mice in the two groups were sacrificed. Samples were rapidly collected and then stored at -80°C. The fecal samples were used for both 16s rRNA analysis and metabolism analysis. Meanwhile, the hippocampus is used for metabolism analysis. In this study, the mice in CSDS group with a social interaction (SI) ratio>=1 were considered stress-resilient and excluded from subsequent experiments (n=4). During the development of CSDS, three mice in CSDS group incurred bite marks: i) two mice (SI ratio <1) were still included in this study; and ii) one mouse (SI ratio >=1) was excluded from this study). The procedure of 16S rRNA gene sequencing analysis was exactly conducted in accordance to our previous studies (Wu et al., 2020; Tian et al., 2022) (**Supplementary File 1**), and the procedures of detecting microbial metabolites in feces using LC-MS were exactly completed according to our previous studies (Wu et al., 2020; Tian et al., 2022) (**Supplementary File 1**).

### Statistical analysis

Student’s t-test, nonparametric Mann-Whitney, or Pearson correlation analysis was used when appropriate. Here, four parameters (ace, chao, shannon and simpson) were calculated to evaluate the alpha diversity of gut microbiota. The principal coordinate analysis (PCoA) was used to assess the beta diversity of gut microbiota. The linear discriminant-analysis (LDA) effect size (LEfSe) was conducted to identify the differential gut microbiota between the two groups, and the phylogenetic investigation of communities by reconstruction of unobserved states (PICRUSt) analysis based on Kyoto Encyclopedia of Genes and Genomes (KEGG) database was performed to predict the potential functions of the differential gut microbiota. To identify the differential microbial metabolites (variable importance in projection (VIP) > 1.0 and p-value<0.05) between the two groups, the orthogonal partial least squares (OPLS) model was built using microbial metabolites. All the analyses was carried out using SPSS 19.0, R software 4.0 and Cytoscape 5.0, and p<0.05 was considered to be statistically significant.

## Results

### CSDS-induced depressive-like behaviors

**Figure 1:**
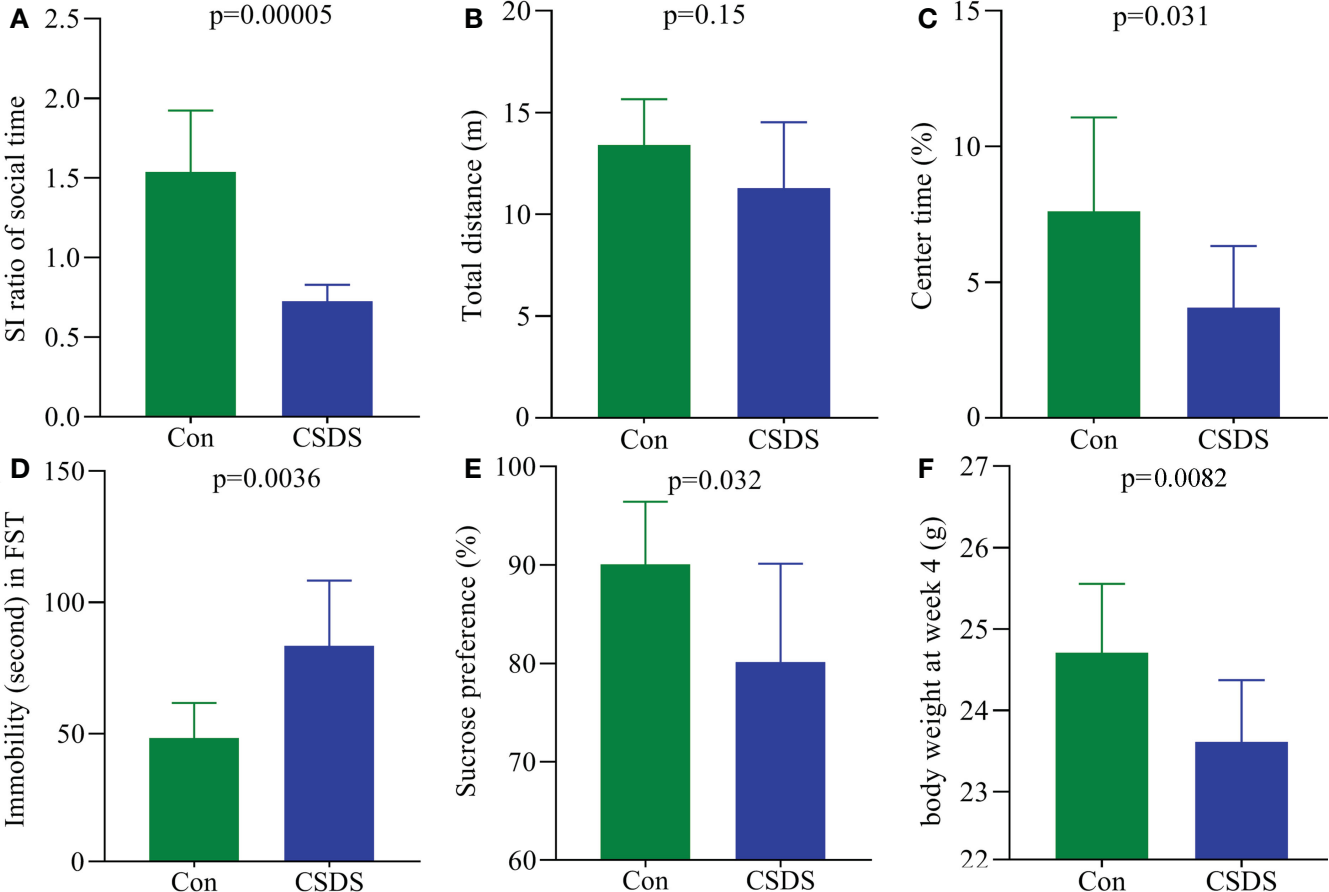
Depressive-like behaviors in CSDS-induced depressed mice. **(A)** CSDS mice had the significantly lower SI ratio compared to control mice; **(B, C)** in open field test, CSDS mice had the similar total distance **(B)** and significantly lower center time (%) **(C)** compared to control mice; **(D)** the immobility time was significantly higher in CSDS mice than in control mice; **(E, F)** after CSDS procedure, both sucrose preference (%) **(E)** and body weight **(F)** were significantly lower in CSDS mice than in control mice. SI, social interaction; Con, control; CSDS, chronic social defeat stress; FST, force swimming test.

As shown in **Figure 1A**, CSDS group showed a significantly lower SI ratio of social time in SIT compared to control group (p=0.00005), indicating that the mice in CSDS group had the social interaction deficiency behavior. In OFT, the total distance was similar between the two groups (**Figure 1B**), indicating the comparable motor functions between control mice and CSDS mice; but the CT(%) was significantly lower in CSDS group than in control group (**Figure 1C**, p=0.031). In FST, CSDS group showed the significantly higher IT compared to control group (**Figure 1D**, p=0.0036). During the whole CSDS, the food intake between the two groups was not statistically different. After the CSDS procedure, significant differences in both SPT (**Figure 1E**, p=0.032) and BW (**Figure 1F**, p=0.0082) were found between the two groups. These results showed that the mice in CSDS group had the depressive-like behaviors, demonstrating that CSDS-induced depression model was successfully established.

### Differential gut microbiota

**Figure 2:**
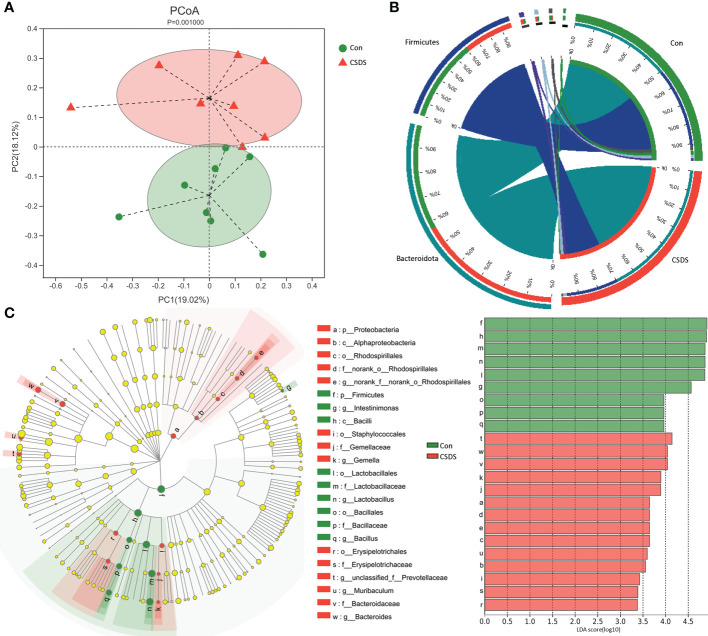
Differential gut microbiota composition between the two groups. **(A)** principal coordinate analysis (PCoA) showed an obvious difference in gut microbiota composition between the two groups; **(B)** the dominant bacteria taxa on phylum level in both groups were Firmicutes and Bacteroidota; **(C)** the linear discriminant-analysis effect size (LEfSe) showed that there were 23 differential bacterial taxa between the two groups, and most of them (n=13) belonged to phylum Firmicutes. Con, control; CSDS, chronic social defeat stress; LDA, linear discriminant-analysis.

The results of within-sample (α) phylogenetic diversity analysis showed no significant differences on alpha diversity between the two groups (ace, p=0.64; chao, p=0.64; Shannon, p=0.11; simpson, p=0.09). But the results of PCoA showed that there were significant differences on gut microbiota compositions between the two group (**Figure 2A**, p=0.0010). The relative abundance on the phylum level was described in **Figure 2B**, and the dominant bacteria taxa on phylum level in both groups were Firmicutes and Bacteroidota. To identify the bacterial taxa responsible for discriminating CSDS mice from control mice, the LEfSe was used here. The bacterial taxa with LDA>2.0 was identified as the differential bacterial taxa. The results showed that there were 24 differential bacterial taxa between the two groups were found (**Figure 2C**). The 14 (60.87%) of these differential bacterial taxa belonged to phylum Firmicutes. The detailed information of these differential bacterial taxa was described in **Supplementary Table S1**.

### Function predictions of differential bacterial taxa

**Figure 3:**
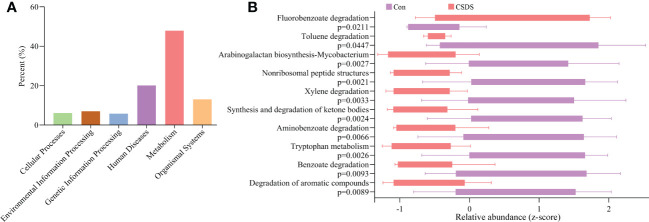
Functional predictions of the differential bacterial taxa. **(A)** the phylogenetic investigation of communities by reconstruction of unobserved states (PICRUSt) analysis showed that the metabolism category ranked the top, with a proportion of 47.92%; **(B)** in the metabolism category, ten significantly affected metabolism pathways were identified.

To find out the potential functions that these differential bacterial taxa were involved in, we used PICRUSt to predict the abundance of functional categories based on the standardized OTU abundance and KEGG database. The results showed that microbial gene functions related to ‘Metabolism’ was the most, accounting for 47.92% of the functional categories in the first level of KEGG pathways (**Figure 3A**). In the second level of KEGG pathways, we found that amino acid metabolism and lipid metabolism were the top five metabolic pathways among ‘Metabolism’ category. Further analysis found that there were 10 significantly affected metabolic pathways (the third level of KEGG pathways) in CSDS mice (**Figure 3B**), and tryptophan metabolism was significantly decreased in CSDS mice (p=0.0026).

### Correlations between differential bacterial taxa and depressive-like behaviors

**Figure 4:**
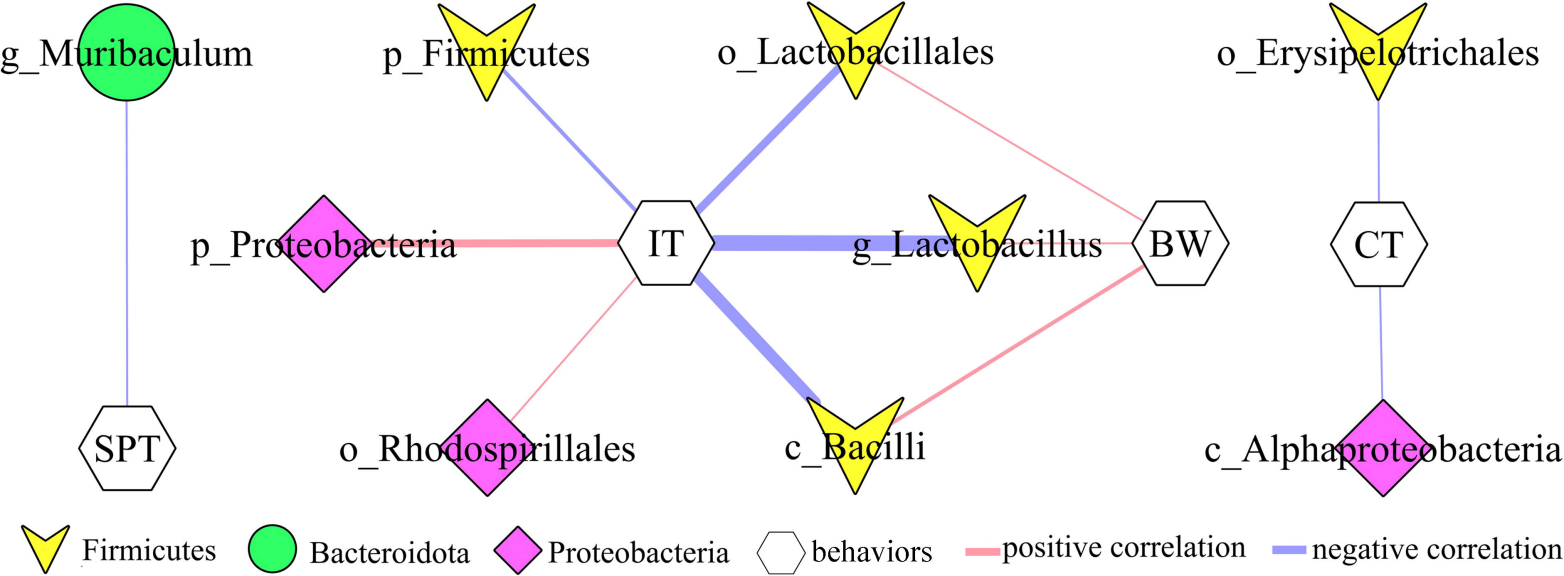
Correlations between differential bacterial taxa and depressive-like behaviors. SPT, sucrose preference; IT, immobility time; CT, center time (%); BW, body weight.

In addition, to explore the potential correlations between differential bacterial taxa and depressive-like behaviors, Pearson correlation analysis was used here. The results showed that there were close relationships between depressive-like behaviors and nine differential bacterial taxa (55.55% belonged to phylum Firmicutes): i) IT was significantly correlated with six differential bacterial taxa; ii) BW was significantly correlated with three differential bacterial taxa under phylum Firmicutes; iii) SPT was significantly correlated with genus Muribaculum under phylum Bacteroidota; and iv) CT was significantly correlated with two differential bacterial taxa (**Figure 4**).

### Differential microbial metabolites

**Figure 5:**
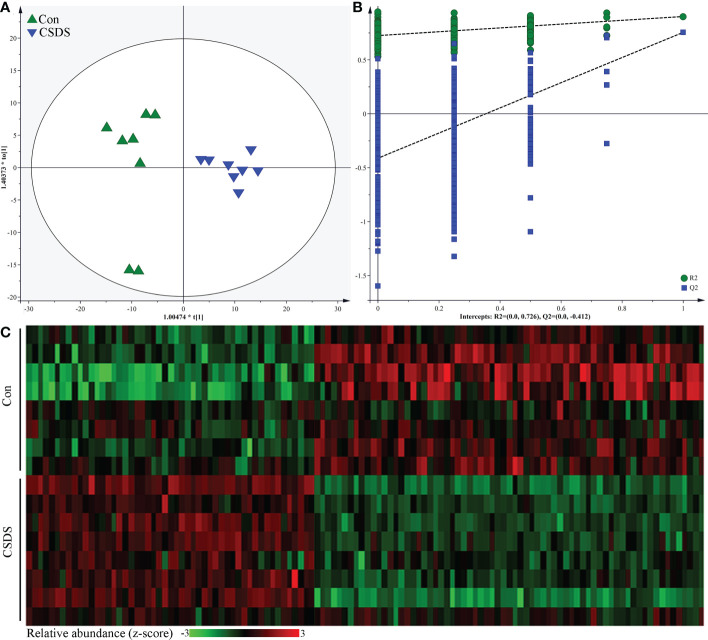
Differential microbial metabolites in CSDS mice. **(A)** the orthogonal partial least squares (OPLS) model showed that the two groups were significantly separated, indicating the divergent microbial metabolic phenotypes in CSDS mice; **(B)** the results of 399-permutation test suggested that the built OPLS model was valid and not over-fitting; **(C)** heat-map of the 120 identified differential metabolites.

The built OPLS model showed that the mice in CSDS group was significantly separated from mice in control group, indicating that there were divergent microbial metabolic phenotypes between CSDS group and control group (**Figure 5A**). Meanwhile, the results of 399-permutation test showed that the regression line of the Q2-points intersected the vertical axis below zero (Q2=-0.412), demonstrating the valid and not over-fitting of this model (**Figure 5B**). By analyzing the corresponding loading plots, 120 differential microbial metabolites were identified (VIP>1.0 and p-value<0.05). Among these microbial metabolites, 51 and 69 metabolites were significantly increased and decreased, respectively, in CSDS group than in control group. These differential microbial metabolites mainly belonged to lipid-related metabolism and amino acid-related metabolism. The heat-map built with the relative abundances of differential microbial metabolites showed a consistent clustering pattern within the individual groups (**Figure 5C**). The detailed information of these differential microbial metabolites was described in **Supplementary Table S2**. The results of Pearson correlation analysis indicated that depressive-like behaviors were mainly significantly correlated with differential microbial metabolites belonged to lipid-related metabolism and amino acid-related metabolism (**Supplementary Figure S1**).

### Tryptophan metabolism as bridge between gut and brain

**Figure 6:**
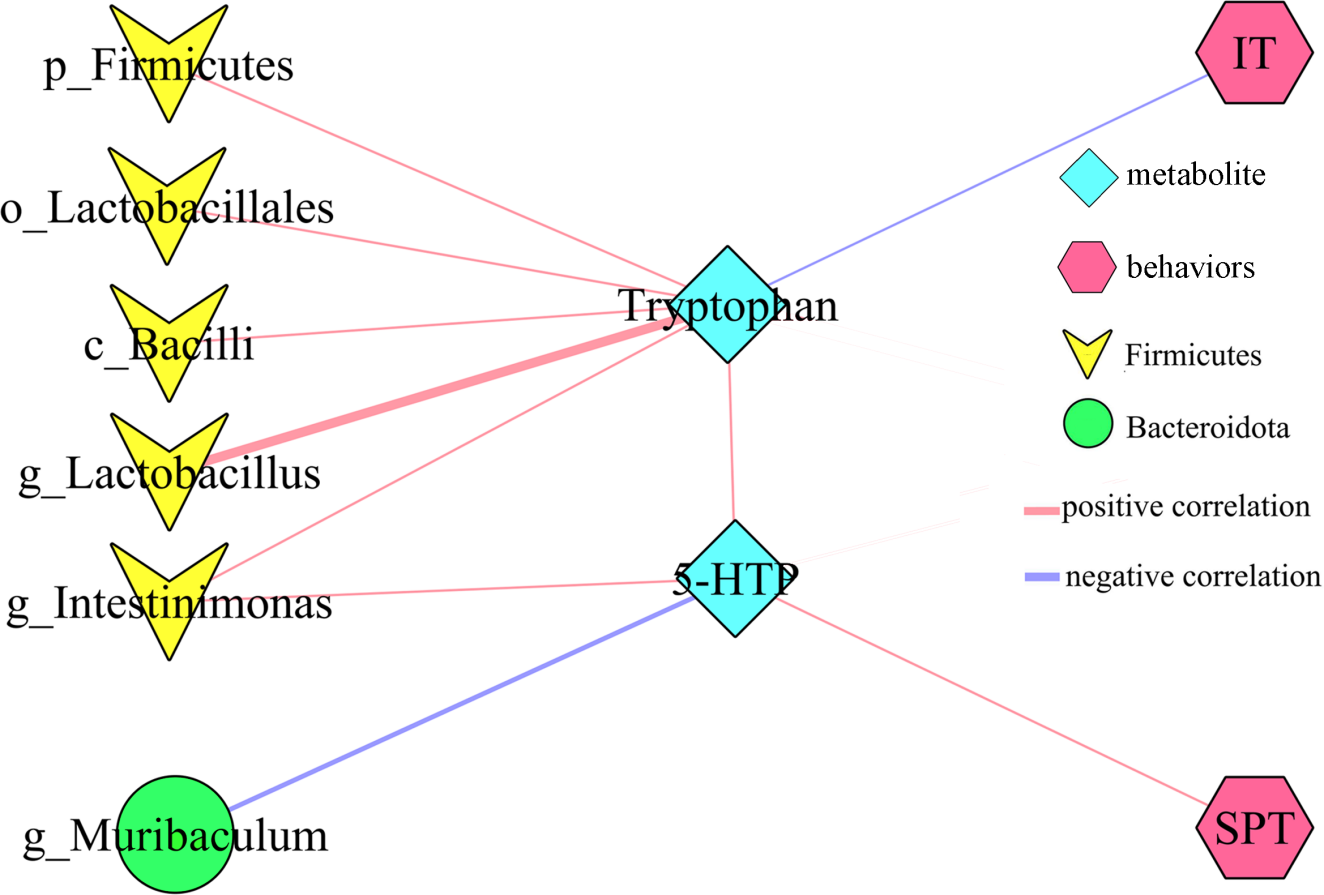
Correlations between differential bacterial taxa, 5-HTP, Tryptophan and depressive-like behaviors. SPT, sucrose preference; IT, immobility time; 5-HTP, 5-hydroxytryptophan.

**Figure 7:**
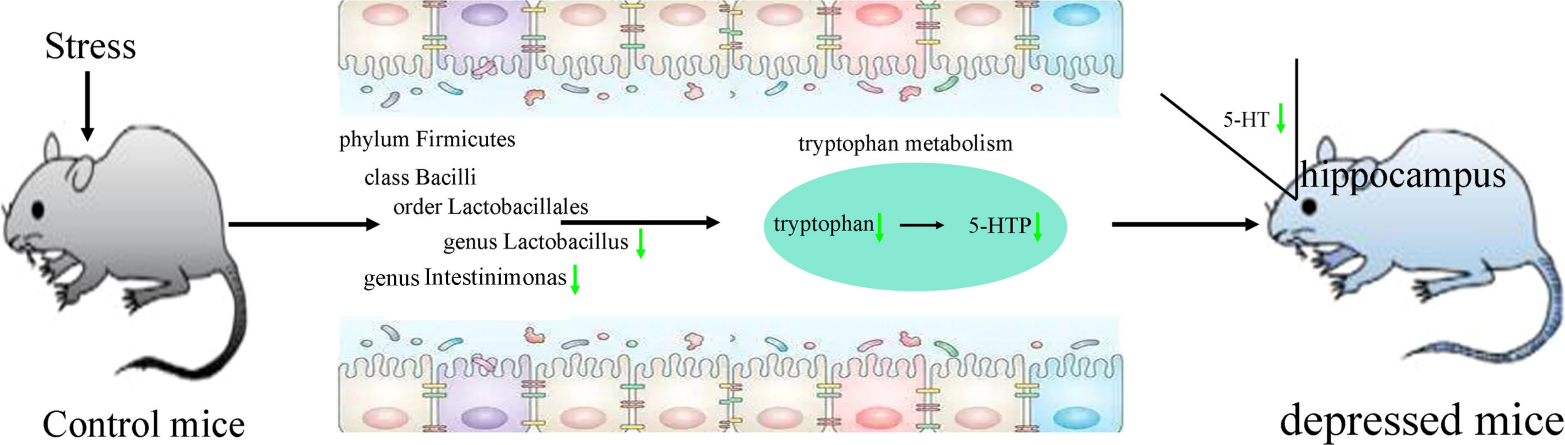
Tryptophan metabolism acting as a bridge in the crosstalk of gut and brain. 5-HTP, 5-hydroxytryptophan; 5-HT, 5-hydroxytryptamine.

PICRUSt functional prediction of the disordered gut microbiota showed that tryptophan metabolism was significantly decreased in this study. Meanwhile, two metabolites (5-hydroxytryptophan and tryptophan) in tryptophan metabolism were found to be significantly decreased in feces of depressed mice here. The results of correlation analysis indicated that there were strong correlations between tryptophan and differential bacterial taxa under Firmicutes, especially genus *Lactobacillus* (r=0.801, p=0.0002) (**Figure 6**), suggested that the disorder of tryptophan metabolism was closely related to the disturbance of phylum Firmicutes. Significantly correlations also existed between tryptophan and IT (r=-0.540, p=0.031), 5-hydroxytryptophan (5-HTP) and SPT (r=-0.562, p=0.023), genus Muribaculum and 5-HTP (r=-0.632, p=0.009). The detailed information of correlations was described in **Supplementary Table S3**. In addition, we found that there was significantly decreased level of 5-hydroxytryptamine (5-HT) in hippocampus of depressed mice (p=0.015). Thus, we suggested that tryptophan metabolism might act as a bridge in the crosstalk of gut and brain (**Figure 7**).

## Discussion

As a psychosocial stress model based on social conflict, CSDS has been widely used in depression studies (Lu et al., 2021; Liu et al., 2022). In the present study, we observed that the mice in the CSDS group showed obvious depressive-like behaviors, suggesting that the depression mice model was successfully established. By 16S rRNA gene sequencing analysis, we identified 24 differential bacterial taxa in CSDS mice, and most of them belonged to phylum Firmicutes. By LC-MS, we found 120 differential microbial metabolites, which mainly belonged to lipid-related metabolism and amino acid-related metabolism. Correlation analysis showed that depressive-like behaviors were closely related to differential bacterial taxa under phylum Firmicutes and differential microbial metabolites that mainly belonged to lipid-related metabolism and amino acid-related metabolism. Our results would be helpful for future exploring the role of gut microbiota in the pathogenesis of depression.

The homeostasis of gut microbiota is important for host’s health, and its disturbances were related with many diseases (Li et al., 2022; Li et al., 2022; Zhang et al., 2022). Firmicutes are bacterial phyla that dominate the entire human digestive tract, and its disturbance is usually viewed as one of hallmark in depression. But the specific molecular mechanisms between depression and phylum Firmicutes are still unclear. *Lactobacillus* is one of beneficial bacteria under phylum Firmicutes. Bravo et al. reported that *Lactobacillus* supplementation could regulate the emotional behavior of mice *via* the vagus nerve (Bravo et al., 2011). Dong et al. observed the significantly decreased level of *Lactobacillus* in depressed mice, and its level was restored after treating with antidepressants (Dong et al., 2022). Schaub et al. found that the increase level of *Lactobacillus* was associated with decreased depressive symptoms in depression patients receiving probiotics (Schaub et al., 2022). Here, we found the significantly decreased level of *Lactobacillus* in CSDS group and the strong correlation between *Lactobacillus* and tryptophan. These results highlighted the role of gut mcirobiota in depression and emphasized the potential of microbiota-related treatment approaches for depression.

In this study, we observed the alterations in lipid and amino acid metabolism in CSDS mice. Meanwhile, the significant lower body weight in CSDS mice was found, although the food intake between the two groups was not statistically different. However, it was uncertain whether the lower body weight in CSDS mice was caused by the disturbance of lipid and amino acid metabolism. Using chronic restraint stress-induced depression model, we found the significantly lower body weight and alterations in lipid metabolism in depressed mice (Tian et al., 2022). But using chronic unpredictable mild stress-induced depression model, we observed the similar body weight between the two groups and alterations in lipid metabolism in depressed mice (Xie et al., 2023). Previous studies reported that gut microbiota was also closely involved in regulating body weight homeostasis (Łoniewski et al., 2022; Van Hul and Cani, 2023). These results indicated that the body weight might be related with many factors, such as gut microbiota and host’s metabolism. Future studies were needed to further explore the potential mechanism under the changes of body weight in depressed mice.

Forced swim test was one of the classic behavioral tests for depression, and immobility time was the evaluation indicator. Here, the significantly higher immobility time was observed in CSDS mice compared to control mice. Anhedonia was one of core symptoms of depression, and sucrose preference test was the most frequently used method for measuring anhedonia. In this study, we identified the significantly lower sucrose preference in CSDS mice compared to control mice. Similar results about immobility time and sucrose preference between depressed mice and control mice were also observed in our previous studies (Tian et al., 2022; Xie et al., 2023). In one study, we found that the significantly decreased level of 5-HT, one of neurotransmitter in tryptophan metabolism, was significantly correlated with immobility time and sucrose preference (Tian et al., 2022). Meanwhile, we also found that two main neurotransmitters in tryptophan metabolism (tryptophan and 5-HT) were significantly decreased in plasma of depression patients (Pan et al., 2018). These results suggested that tryptophan metabolism might have an important role in the onset of depressive-like behaviors.

Tryptophan is the sole precursor of 5-HT, which is an important monoamine neurotransmitter. Many studies have reported that the level of 5-HT was significantly decreased in depression (Cai et al., 2022; Zhu et al., 2022). Our previous studies also found the significantly decreased level of 5-HT in depression patients’ plasma and brain areas of depressed mice (Wu et al., 2020; Tian et al., 2022; Xie et al., 2023). 5-HT is mainly produced in gut, but it cannot cross the blood-brain barrier. However, its direct biosynthetic precursor, 5-HTP can cross blood-brain barrier. In this study, we found the significantly decreased levels of tryptophan and 5-HTP in feces of depressed mice. Considering the decreased level of 5-HT in hippocampus, we deduced that the disordered gut microbiota, especially *Lactobacillus*, resulted in the significantly decreased tryptophan metabolism, which caused the decreased level of 5-HTP; then the decreased 5-HTP level caused the lower level of 5-HT in hippocampus, and eventually the mice showed depressive-like behaviors. These results demonstrated that tryptophan metabolism might be a bridge between gut microbiota and brain in depressed mice.

## Conclusion

In conclusion, the CSDS successfully induced depressive-like behaviors in mice, and the depressed mice showed significantly different gut microbiota compositions compared to control mice. Most of the differential bacterial taxa belonged to phylum Firmicutes. Meanwhile, there were divergent microbial metabolic phenotypes between control mice and CSDS mice. The identified differential microbial metabolites mainly belonged to lipid-related metabolism and amino acid-related metabolism. Further analysis showed that tryptophan metabolism was significantly decreased, and there was strong correlation between genus *Lactobacillus* under Firmicutes and tryptophan. Considering the significantly decreased level of 5-HTP in feces and 5-HT in hippocampus, our findings indicated that Firmicutes, especially genus *Lactobacillus*, might play a key role in the onset of depression *via* tryptophan metabolism.

## Data availability statement

The datasets presented in this study can be found in online repositories. The names of the repository/repositories and accession number(s) can be found below: https://www.ncbi.nlm.nih.gov/bioproject/PRJNA909169.

## Ethics statement

This study was performed according to the National Institutes of Health’s Animal Research Guide, and Ethics Committee of Chongqing Medical University reviewed and approved this study.

## Author contributions

YW, JX, W-TW and J-JC performed material preparation, data collection and analysis. JX, W-TW and J-JC wrote the first draft of the manuscript. QZ, DW, LN, SW and YZ performed model built, software and methodology. YW conducted writing-reviewing and editing. All authors contributed to the article and approved the submitted version.

## References

[B1] Affatato O. Moulin T. C. Pisanu C. Babasieva V. S. Russo M. Aydinlar E. I.. (2021). High efficacy of onabotulinumtoxinA treatment in patients with comorbid migraine and depression: a meta-analysis. J. Transl. Med. 19 (1), 133. doi: 10.1186/s12967-021-02801-w 33789668 PMC8011097

[B2] Bai S. Xie J. Bai H. Tian T. Zou T. Chen J. J. (2021). Gut microbiota-derived inflammation-related serum metabolites as potential biomarkers for major depressive disorder. J. Inflammation Res. 14, 3755–3766. doi: 10.2147/JIR.S324922 PMC8354734 34393496

[B3] Bravo J. A. Forsythe P. Chew M. V. Escaravage E. Savignac H. M. Dinan T. G.. (2011). Ingestion of lactobacillus strain regulates emotional behavior and central GABA receptor expression in a mouse *via* the vagus nerve. Proc. Natl. Acad. Sci. U S A. 108 (38), 16050–16055. doi: 10.1073/pnas.1102999108 21876150 PMC3179073

[B4] Cai W. Wang X. F. Wei X. F. Zhang J. R. Hu C. Ma W.. (2022). Does urinary metabolite signature act as a biomarker of post-stroke depression? Front. Psychiatry 13, 928076. doi: 10.3389/fpsyt.2022.928076 36090365 PMC9448878

[B5] Cai T. Zheng S. P. Shi X. Yuan L. Z. Hu H. Zhou B.. (2022). Therapeutic effect of fecal microbiota transplantation on chronic unpredictable mild stress-induced depression. Front. Cell Infect. Microbiol. 12, 900652. doi: 10.3389/fcimb.2022.900652 35967846 PMC9366333

[B6] Carlessi A. S. Borba L. A. Zugno A. I. Quevedo J. Réus G. Z. (2021). Gut microbiota-brain axis in depression: The role of neuroinflammation. Eur. J. Neurosci. 53 (1), 222–235. doi: 10.1111/ejn.14631 31785168

[B7] Chen J. J. He S. Fang L. Wang B. Bai S. J. Xie J.. (2020). Age-specific differential changes on gut microbiota composition in patients with major depressive disorder. Aging (Albany NY). 12 (3), 2764–2776. doi: 10.18632/aging.102775 32040443 PMC7041727

[B8] Chojnacki C. Konrad P. Błońska A. Medrek-Socha M. Przybylowska-Sygut K. Chojnacki J.. (2022). Altered tryptophan metabolism on the kynurenine pathway in depressive patients with small intestinal bacterial overgrowth. Nutrients 14 (15), 3217. doi: 10.3390/nu14153217 35956393 PMC9370164

[B9] Dong Z. Xie Q. Xu F. Shen X. Hao Y. Li J.. (2022). Neferine alleviates chronic stress-induced depression by regulating monoamine neurotransmitter secretion and gut microbiota structure. Front. Pharmacol. 13, 974949. doi: 10.3389/fphar.2022.974949 36120376 PMC9479079

[B10] Gong X. Huang C. Yang X. Chen J. Pu J. He Y.. (2021). Altered fecal metabolites and colonic glycerophospholipids were associated with abnormal composition of gut microbiota in a depression model of mice. Front. Neurosci. 15, 701355. doi: 10.3389/fnins.2021.701355 34349620 PMC8326978

[B11] Grover S. Adarsh H. (2022). A comparative study of prevalence of mixed features in patients with unipolar and bipolar depression. Asian J. Psychiatr. 81, 103439. doi: 10.1016/j.ajp.2022.103439 36645972

[B12] He L. Zheng Y. Huang L. Ye J. Ye Y. Luo H.. (2022). Nrf2 regulates the arginase 1+ microglia phenotype through the initiation of TREM2 transcription, ameliorating depression-like behavior in mice. Transl. Psychiatry 12 (1), 459. doi: 10.1038/s41398-022-02227-y 36316319 PMC9622811

[B13] Huang F. Wu X. (2021). Brain neurotransmitter modulation by gut microbiota in anxiety and depression. Front. Cell Dev. Biol. 9, 649103. doi: 10.3389/fcell.2021.649103 33777957 PMC7991717

[B14] Jiang W. Gong L. Liu F. Ren Y. Mu J. (2021). Alteration of gut microbiome and correlated lipid metabolism in post-stroke depression. Front. Cell Infect. Microbiol. 11, 663967. doi: 10.3389/fcimb.2021.663967 33968807 PMC8100602

[B15] Li Y. Han M. Song J. Liu S. Wang Y. Su X.. (2022). The prebiotic effects of soluble dietary fiber mixture on renal anemia and the gut microbiota in end-stage renal disease patients on maintenance hemodialysis: A prospective, randomized, placebo-controlled study. J. Transl. Med. 20 (1), 599. doi: 10.1186/s12967-022-03812-x 36517799 PMC9753397

[B16] Li X. Li R. Ji B. Zhao L. Wang J. Yan T. (2022). Integrative metagenomic and metabolomic analyses reveal the role of gut microbiota in antibody-mediated renal allograft rejection. J. Transl. Med. 20 (1), 614. doi: 10.1186/s12967-022-03825-6 36564805 PMC9784291

[B17] Liu X. Gu X. H. Zheng L. L. Xu L. J. Yang Y. J. Yang G.. (2022). Autophagy promotes membrane trafficking of NR2B to alleviate depression by inhibiting AQP4 expression in mice. Exp. Cell Res. 419 (1), 113298. doi: 10.1016/j.yexcr.2022.113298 35961389

[B18] Liu Y. Wang H. Gui S. Zeng B. Pu J. Zheng P.. (2021). Proteomics analysis of the gut-brain axis in a gut microbiota-dysbiosis model of depression. Transl. Psychiatry 11 (1), 568. doi: 10.1038/s41398-021-01689-w 34744165 PMC8572885

[B19] Łoniewski I. Szulińska M. Kaczmarczyk M. Podsiadło K. Styburski D. Skonieczna-Żydecka K.. (2022). Analysis of correlations between gut microbiota, stool short chain fatty acids, calprotectin and cardiometabolic risk factors in postmenopausal women with obesity: A cross-sectional study. J. Transl. Med. 20 (1), 585. doi: 10.1186/s12967-022-03801-0 36503483 PMC9743526

[B20] Lu J. Gong X. Yao X. Guang Y. Yang H. Ji R.. (2021). Prolonged chronic social defeat stress promotes less resilience and higher uniformity in depression-like behaviors in adult male mice. Biochem. Biophys. Res. Commun. 553, 107–113. doi: 10.1016/j.bbrc.2021.03.058 33765554

[B21] Lu C. Wei Z. Wang Y. Li S. Tong L. Liu X.. (2022). Soy isoflavones alleviate lipopolysaccharide-induced depressive-like behavior by suppressing neuroinflammation, mediating tryptophan metabolism and promoting synaptic plasticity. Food Funct. 13 (18), 9513–9522. doi: 10.1039/D2FO01437H 35993820

[B22] Mojtabavi H. Shaka Z. Momtazmanesh S. Ajdari A. Rezaei N. (2022). Circulating brain-derived neurotrophic factor as a potential biomarker in stroke: A systematic review and meta-analysis. J. Transl. Med. 20 (1), 126. doi: 10.1186/s12967-022-03312-y 35287688 PMC8919648

[B23] Ning H. Zhou H. Ren J. Zhou G. Yang N. Wang Z.. (2022). Zishen pingchan granules combined with pramipexole in the improvement of depressive symptoms in parkinson's disease: A prospective, multicenter, randomized, double-blind, controlled clinical study. J. Transl. Med. 20 (1), 357. doi: 10.1186/s12967-022-03551-z 35962349 PMC9373440

[B24] Pan J. X. Xia J. J. Deng F. L. Liang W. W. Wu J. Yin B. M.. (2018). Diagnosis of major depressive disorder based on changes in multiple plasma neurotransmitters: A targeted metabolomics study. Transl. Psychiatry 8 (1), 130. doi: 10.1038/s41398-018-0183-x 29991685 PMC6039504

[B25] Pu J. Liu Y. Gui S. Tian L. Yu Y. Wang D.. (2022). Effects of pharmacological treatment on metabolomic alterations in animal models of depression. Transl. Psychiatry 12 (1), 175. doi: 10.1038/s41398-022-01947-5 35487889 PMC9055046

[B26] Pu Y. Zhang Q. Tang Z. Lu C. Wu L. Zhong Y.. (2022). Fecal microbiota transplantation from patients with rheumatoid arthritis causes depression-like behaviors in mice through abnormal T cells activation. Transl. Psychiatry 12 (1), 223. doi: 10.1038/s41398-022-01993-z 35650202 PMC9160267

[B27] Schaub A. C. Schneider E. Vazquez-Castellanos J. F. Schweinfurth N. Kettelhack C. Doll J. P. K.. (2022). Clinical, gut microbial and neural effects of a probiotic add-on therapy in depressed patients: A randomized controlled trial. Transl. Psychiatry 12 (1), 227. doi: 10.1038/s41398-022-01977-z 35654766 PMC9163095

[B28] Song J. Zhou B. Kan J. Liu G. Zhang S. Si L.. (2022). Gut microbiota: Linking nutrition and perinatal depression. Front. Cell Infect. Microbiol. 12, 932309. doi: 10.3389/fcimb.2022.932309 36093196 PMC9459161

[B29] Tian T. Mao Q. Xie J. Wang Y. Shao W. H. Zhong Q.. (2022). Multi-omics data reveals the disturbance of glycerophospholipid metabolism caused by disordered gut microbiota in depressed mice. J. Adv. Res. 39, 135–145. doi: 10.1016/j.jare.2021.10.002 35777903 PMC9263645

[B30] Tian T. Qin Y. Wu M. Wang W. Song T. Deng X.. (2023). Differential gut microbiota and microbial metabolites in adolescents with depression. Asian J. Psychiatr. 83, 103496. doi: 10.1016/j.ajp.2023.103496 36764124

[B31] Van Hul M. Cani P. D. (2023). The gut microbiota in obesity and weight management: Microbes as friends or foe? Nat. Rev. Endocrinol. 17. doi: 10.1038/s41574-022-00794-0 36650295

[B32] Wang W. Guo H. Zhang S. X. Li J. Cheng K. Bai S. J.. (2016). Targeted metabolomic pathway analysis and validation revealed glutamatergic disorder in the prefrontal cortex among the chronic social defeat stress mice model of depression. J. Proteome Res. 15 (10), 3784–3792. doi: 10.1021/acs.jproteome.6b00577 27599184

[B33] Wu M. Tian T. Mao Q. Zou T. Zhou C. J. Xie J.. (2020). Associations between disordered gut microbiota and changes of neurotransmitters and short-chain fatty acids in depressed mice. Transl. Psychiatry 10 (1), 350. doi: 10.1038/s41398-020-01038-3 33067412 PMC7567879

[B34] Xiao W. Li J. Gao X. Yang H. Su J. Weng R.. (2022). Involvement of the gut-brain axis in vascular depression *via* tryptophan metabolism: A benefit of short chain fatty acids. Exp. Neurol. 358, 114225. doi: 10.1016/j.expneurol.2022.114225 36100045

[B35] Xie J. Wang Y. Zhong Q. Bai S. J. Zhou C. J. Tian T.. (2022). Associations between disordered microbial metabolites and changes of neurotransmitters in depressed mice. Front. Cell Infect. Microbiol. 12, 906303. doi: 10.3389/fcimb.2022.906303 35669116 PMC9163491

[B36] Xie J. Zhong Q. Wu W. T. Chen J. J. (2023). Multi-omics data reveals the important role of glycerophospholipid metabolism in the crosstalk between gut and brain in depression. J. Transl. Med. 21 (1), 93. doi: 10.1186/s12967-023-03942-w 36750892 PMC9903503

[B37] Zhang P. Tang A. Geng Y. Lai J. Gao X. Pan Y.. (2022). Gut microbial trajectory in patients with bipolar depression: A longitudinal study. Asian J. Psychiatr. 73, 103098. doi: 10.1016/j.ajp.2022.103098 35430495

[B38] Zheng P. Zeng B. Zhou C. Liu M. Fang Z. Xu X.. (2016). Gut microbiome remodeling induces depressive-like behaviors through a pathway mediated by the host's metabolism. Mol. Psychiatry 21 (6), 786–796. doi: 10.1038/mp.2016.44 27067014

[B39] Zhong Q. Chen J. J. Wang Y. Shao W. H. Zhou C. J. Xie P. (2022). Differential gut microbiota compositions related with the severity of major depressive disorder. Front. Cell Infect. Microbiol. 12, 907239. doi: 10.3389/fcimb.2022.907239 35899051 PMC9309346

[B40] Zhu Y. He J. Wu C. Wu J. Cheng Z. Chen Y.. (2022). Transcranial ultrasound stimulation relieves depression in mice with chronic restraint stress. J. Neural Eng. doi: 10.1088/1741-2552/ac8bfd 35998565

