# Supplementary material for: Tryptophan metabolism as bridge between gut microbiota and brain in chronic social defeat stress-induced depression mice
Source: Front Cell Infect Microbiol. 2023 Feb 24;13:1121445. doi: 10.3389/fcimb.2023.1121445 (PMC9999000; doi:10.3389/fcimb.2023.1121445)
Supplement: Supplementary file 1 [file DataSheet_1.docx]

**Supplementary information**

***1. Behaviors testing methods***

Briefly, in open field test (OFT), we put the mice into the center of box (45 x 45 x 45 cm) to freely explore the box for six minutes. The data produced in last five minutes were collected, including total distance, center time and center distance. In forced swim test (FST), we put the mice into a plexigas cylinder (15 cm diameter x 30 cm height) for six minutes. The plexiglas cylinder was 15 cm diameter and 30 cm height and filled with 18 cm height of water. The temperature of water was 24 ±1°C. The immobility time in last five minutes was collected. In sucrose preference test, firstly, the mice were trained to adapt to 1% sucrose solution before test; secondly, after adaption, the mice were freely access to 1% sucrose solution or water. The consumptions of 1% sucrose solution and water were collected. The sucrose preference was defined as the proportion of 1% sucrose solution consumption in the total liquid consumptions.

***2. 16S rRNA gene sequencing***

Total DNA was extracted from fecal samples using the E.Z.N.A.® soil DNA Kit (Omega Bio-tek, Norcross, GA, U.S.), according to the manufacturer’s protocol. All DNA samples were quality checked and the concentration was quantified by NanoDrop 2000 spectrophotometers (Thermo Fisher Scientific, Wilmington, DE, USA). Bacterial 16S rRNA gene fragments (V3-V4) were amplified from the extracted DNA using primers 338F (5'-ACTCCTACGGGAGGCAGCAG-3') and 806R(5'-GGACTACHVGGGTWTCTAAT-3'), and the following PCR conditions: 30 s at 95 °C, 30 s at 55 °C, and 45 s at 72 °C for 27 cycles. PCRs were performed with 4 μL 5 × TransStart FastPfu buffer, 2 μL 2.5 mM deoxynucleoside triphosphates (dNTPs)，0.8 μL of each primer (5 μM), 0.4 μL TransStart FastPfu DNA Polymerase, 10 ng of extracted DNA, and finally using ddH2O to make up 20 μL. Agarose gel electrophoresis was performed to verify the size of amplicons. Amplicons were subjected to paired-end sequencing on the Illumina MiSeq sequencing platform using PE300 chemical at Majorbio Bio-Pharm Technology Co. Ltd. (Shanghai, China).

After demultiplexing, the resulting sequences were merged with FLASH (v1.2.11) and quality filtered with fastp (0.19.6). Then the high-quality sequences were de-noised using DADA2 plugin in the Qiime2 pipeline with ecommended parameters, which obtains single nucleotide resolution based on error profiles within samples. DADA2 denoised sequences are usually called amplicon sequence variants (ASVs). To minimize the effects of sequencing depth on alpha and beta diversity measure, the number of sequence from each sample was rarefied to 4000, which still yielded an average Good’s coverage of 97.90%. Taxonomic assignment of ASVs was performed using the Naive bayes consensus taxonomy classifier implemented in Qiime2 and the SILVA 16S rRNA database (v138). Analyses of the 16S rRNA microbiome sequencing data were performed using the free online platform of Majorbio Cloud Platform (www.majorbio.com).

***3. LC-MS procedure***

Briefly, the LC-MS procedure was as following. The chromatographic condition: the samples were separated by Agilent 1290 infinity LC ultra high performance liquid chromatography system (UHPLC) HILIC and C18 chromatographic columns; the column temperature of HILIC chromatographic column was 35°C, the flow rate was 0.3ml/min and the injection volume was 2 μl; Mobile phase composition A: 90% water + 2 mM ammonium formate + 10% acetonitrile, B: methanol + 0.4% formic acid; The gradient elution procedure was as follows: 0-1.0 min, 85% B; 1.0-3.0min, B changeed linearly from 85% to 80%; 3.0-4.0 min, 80% B; 4.0-6.0 min, B changed linearly from 80% to 70%; 6.0-10.0 min, B changed linearly from 70% to 50%; 10-12.5 min, B maintained at 50%; 12.5-12.6 min, B changed linearly from 50% to 85%; 12.6-18min, B maintained at 85%. C18 chromatographic column temperature was 40°C, flow rate was 0.4ml/min and injection volume was 2 μl; Mobile phase composition A: water + 5 mM ammonium acetate + 0.2% ammonia, B: 99.5% acetonitrile + 0.5% ammonia; The gradient elution procedure was as follows: 0-5 min, B changed linearly from 5% to 60%; 5-11 min, B changed linearly from 60% to 100%; 11-13 min, B maintained at 100%; 13-13.1 min, B changed linearly from 100% to 5%; 13.1-16min, B maintained at 5%; During the whole analysis process, the samples were placed in a 4°C automatic sampler. In order to avoid the influence caused by the fluctuation of instrument detection signal, the random sequence was used for continuous analysis of samples. QC samples were inserted into the sample queue to monitor and evaluate the stability of the system and the reliability of experimental data. The mass spectrometry conditions: AB 6500 qtrap mass spectrometer was used (AB SCIEX) for mass spectrometry analysis. The ESI source conditions are as follows: weather gas temperature, 350°C; dry gas temperature, 350°C; weather gas flow, 11L/ min; dry gas flow, 10 L/min; capacitive voltage, 4000 V or - 3500 V in positive or negative modes, carefully; nozzle voltage, 500 V; and nebulizer pressure, 30 psi, monitored by multiple reaction monitoring (MRM) mode. We used multiquant or analyst software to extract the peak of MRM raw data to obtain the ratio of peak area of each substance to internal standard peak area. Then, we calculated the content of metabolites according to the standard curve.

**Table S1 Differential bacterial taxa between the two groups**

| **phylum** | **group** | **Mean** | **LDA** | **p** | **bacterial taxa** |
| --- | --- | --- | --- | --- | --- |
| p_Bacteroidota | CSDS | 4.38 | 4.04 | 0.01 | f_Bacteroidaceae |
| p_Bacteroidota | CSDS | 4.38 | 4.04 | 0.01 | g_Bacteroides |
| p_Bacteroidota | CSDS | 3.96 | 3.59 | 0.05 | g_Muribaculum |
| p_Bacteroidota | CSDS | 3.84 | 4.14 | 0.01 | g_unclassified_f_Prevotellaceae |
| p_Firmicutes | Con | 5.64 | 4.92 | 0.02 | p_Firmicutes |
| p_Firmicutes | Con | 5.53 | 4.91 | 0.01 | c_Bacilli |
| p_Firmicutes | Con | 4.39 | 3.96 | 0.03 | o_Bacillales |
| p_Firmicutes | Con | 4.39 | 3.96 | 0.03 | f_Bacillaceae |
| p_Firmicutes | Con | 4.39 | 3.96 | 0.03 | g_Bacillus |
| p_Firmicutes | CSDS | 3.69 | 3.38 | 0.01 | o_Erysipelotrichales |
| p_Firmicutes | CSDS | 3.49 | 3.39 | 0.05 | f_Erysipelotrichaceae |
| p_Firmicutes | Con | 5.49 | 4.87 | 0.02 | o_Lactobacillales |
| p_Firmicutes | Con | 5.49 | 4.87 | 0.01 | f_Lactobacillaceae |
| p_Firmicutes | Con | 5.49 | 4.87 | 0.01 | g_Lactobacillus |
| p_Firmicutes | CSDS | 3.54 | 3.43 | 0.02 | o_Staphylococcales |
| p_Firmicutes | CSDS | 2.66 | 3.89 | 0.01 | f_Gemellaceae |
| p_Firmicutes | CSDS | 2.66 | 3.89 | 0.01 | g_Gemella |
| p_Firmicutes | Con | 2.34 | 4.57 | 0.01 | g_Intestinimonas |
| p_Proteobacteria | CSDS | 3.96 | 3.64 | 0.02 | p_Proteobacteria |
| p_Proteobacteria | CSDS | 3.19 | 3.56 | 0.02 | c_Alphaproteobacteria |
| p_Proteobacteria | CSDS | 3.09 | 3.64 | 0.03 | o_Rhodospirillales |
| p_Proteobacteria | CSDS | 3.09 | 3.64 | 0.03 | f_norank_o_Rhodospirillales |
| p_Proteobacteria | CSDS | 3.09 | 3.64 | 0.03 | g_norank_f_norank_o_Rhodospirillales |

**Table S2 Differential microbial metabolites between the two groups**

| **Metabolite** | **VIP** | **P** | **FC** |
| --- | --- | --- | --- |
| 4a-Methylzymosterol-4-carboxylic acid | 1.06 | 0.00124 | 0.91 |
| Gibberellin A37 | 1.23 | 0.00202 | 1.09 |
| Glycerol 3-phosphate | 1 | 0.0193 | 0.81 |
| Uridine | 1.01 | 0.04956 | 0.95 |
| Cysteine-S-sulfate | 1 | 0.0357 | 1.02 |
| Homoveratric acid | 1.06 | 0.00884 | 0.95 |
| 5,8,11,14-Icosatetraenoic Acid | 1.06 | 0.00953 | 0.93 |
| Orotidine | 1.17 | 0.00051 | 0.84 |
| D-Myoinositol 4-phosphate | 1.04 | 0.03112 | 0.91 |
| D-Glucose 6-phosphate | 1.03 | 0.03277 | 0.9 |
| 1-(sn-Glycero-3-phospho)-1D-myo-inositol | 1.11 | 0.00092 | 0.81 |
| Aldosterone 18-glucuronide | 1.08 | 0.00448 | 0.86 |
| Pyrroline hydroxycarboxylic acid | 1.02 | 0.01488 | 1.49 |
| propionic acid | 1.05 | 0.02372 | 1.2 |
| 20-Hydroxyeicosatrieneoic acid | 1.16 | 0.0011 | 1.02 |
| Vanillin acetate | 1.03 | 0.03956 | 0.92 |
| Ramipril Diketopiperazine | 1 | 0.04628 | 0.88 |
| Bilobalide | 1.02 | 0.03893 | 1.31 |
| (Z)-Narceine imide | 1.07 | 0.0366 | 1.12 |
| PE(P-18:0/0:0) | 1.19 | 0.00069 | 0.92 |
| 11-Hydroxyeicosatetraenoate glyceryl ester | 1.06 | 0.02843 | 0.92 |
| Ganoderic acid L | 1.07 | 0.01185 | 1.04 |
| Isorhamnetin-3-oglucoside | 1.06 | 0.00386 | 0.98 |
| PC(16:0/0:0) | 1.02 | 0.00179 | 1.04 |
| LysoPC(P-16:0) | 1.1 | 0.00059 | 0.88 |
| PGA1 | 1.03 | 0.01012 | 0.97 |
| Isovalerylalanine | 1.08 | 0.036 | 1 |
| Neuromedin N (1-4) | 1.07 | 0.00684 | 1.04 |
| LysoPE(18:1(11Z)/0:0) | 1.02 | 0.01261 | 0.92 |
| LPA(20:3n6/0:0) | 1.01 | 0.00027 | 0.99 |
| Stearoylcarnitine | 1.23 | 0.00035 | 0.92 |
| Hydroxybuprenorphine | 1.25 | 0.00018 | 0.82 |
| LysoPE(20:1(11Z)/0:0) | 1.19 | 0.00014 | 0.87 |
| Docosa-4,7,10,13,16,19-hexaenoate | 1.2 | 0.00056 | 0.93 |
| Epoxydocosapentaenoic acid | 1.01 | 0.01187 | 0.92 |
| kainic acid | 1.08 | 0.00537 | 0.98 |
| Succinyldisalicylic acid | 1.08 | 0.00306 | 0.97 |
| 5-Hydroxytryptophan | 1.01 | 0.01259 | 1.25 |
| Apigenin 7-arabinoside | 1.09 | 0.00321 | 0.98 |
| Ethyl (2E,4E,7Z)-decatrienoic acid | 1.05 | 0.00853 | 1.03 |
| 13-HDoHE | 1.14 | 0.0017 | 1.05 |
| 5-Deoxykievitone hydrate | 1.05 | 0.02143 | 0.88 |
| Blumealactone C | 1.1 | 0.0072 | 0.92 |
| (1E,4Z,6a,8b,10a)-8-Angeloyloxy-10,15-dihydroxy-3-oxo-1,4,11(13)-germacratrien-12,6-olide | 1.01 | 0.01728 | 0.93 |
| 2-Methoxyestrone | 1.13 | 0.01171 | 1.2 |
| Gibberellin A95 | 1.04 | 0.02976 | 0.89 |
| Iodotyrosine | 1.05 | 0.02685 | 1.09 |
| (3b,6b,8a,12a)-8,12-Epoxy-7(11)-eremophilene-6,8,12-trimethoxy-3-ol | 1.27 | 0.00035 | 1.02 |
| 2-Carboxy-4-decanolide | 1.21 | 0.0004 | 1.02 |
| 4,6-Decadiyn-1-ol | 1.13 | 0.00222 | 1.02 |
| beta-Costic acid | 1.08 | 0.0239 | 1.05 |
| Isomucronulatol | 1.13 | 0.00185 | 1.14 |
| 6-Phenyl-3-hexen-2-one | 1.1 | 0.0039 | 1.02 |
| 3,4-DHPEA-EA | 1.13 | 0.00504 | 1.08 |
| (1R*,3R*,3'S*)-1,2,3,4-Tetrahydro-1-(2-thio-3-pyrrolidinyl)-beta-carboline-3-carboxylic acid | 1.24 | 0.00048 | 1.11 |
| Ethyl (S)-3-hydroxybutyric acid glucoside | 1.01 | 0.00261 | 0.9 |
| pentanoic acid | 1.01 | 0.03241 | 1.13 |
| 2,3-Dimethyl-3-hydroxyglutaric acid | 1.19 | 0.00022 | 1.06 |
| L-Citronellol glucoside | 1 | 0.04258 | 1.03 |
| Valyl-Proline | 1 | 0.01944 | 0.96 |
| 2',2'-Difluorodeoxyuridine | 1.11 | 0.00672 | 0.87 |
| Acetic acid | 1.03 | 0.01305 | 1.27 |
| Xi-2,3-Dihydro-3,5-dihydroxy-6-methyl-4H-pyran-4-one | 1.04 | 0.02687 | 0.88 |
| DCMP | 1.05 | 0.01793 | 0.92 |
| 12-Hydroxynevirapine glucuronide | 1.05 | 0.00841 | 0.9 |
| (6S)GlcNAcb | 1.23 | 0.00153 | 0.9 |
| 1-Deoxy-D-xylulose | 1.16 | 0.00415 | 0.86 |
| 3-Hydroxy-C4-HSL | 1.14 | 0.01174 | 1.03 |
| N-Acetyl-L-glutamic acid | 1.09 | 0.00449 | 1.07 |
| PA(O-16:0/0:0) | 1.18 | 0.00109 | 0.79 |
| Tryptophan | 1.03 | 0.00348 | 1.31 |
| PE(24:1(15Z)/24:1(15Z)) | 1 | 0.03491 | 1.28 |
| L-Threoneopterin | 1.07 | 0.02604 | 1.21 |
| N-Feruloylglycine | 1.04 | 0.0167 | 1.25 |
| Verbenalin | 1.02 | 0.02987 | 0.85 |
| 15-Octadecene-9,11,13-triynoic acid | 1.11 | 0.0062 | 1.13 |
| 2-(3-Hydroxy-4-methylphenyl)-5-methyl-4-hexen-3-one | 1.27 | 0.00063 | 1.03 |
| 2-Hydrazinopyridine | 1.18 | 0.00532 | 1.02 |
| 24-Hydroxycalcitriol | 1.01 | 0.02004 | 1.02 |
| Coumestan | 1 | 0.00549 | 0.99 |
| Austalide L | 1.03 | 0.01219 | 0.97 |
| Cis-3-Hexenyl tiglate | 1.3 | 0.00011 | 1.02 |
| Octadeca-2,4-dienoic acid | 1.03 | 0.00686 | 1.06 |
| PC(P-18:0/0:0) | 1.03 | 0.00473 | 0.89 |
| PE(P-16:0/0:0) | 1.24 | 0.00002 | 0.79 |
| Phenobarbital | 1.12 | 0.00734 | 1.01 |
| 2-Pyrrolidineacetic acid | 1.13 | 0.00138 | 1.01 |
| PE(P-20:0/0:0) | 1.14 | 0.00163 | 0.86 |
| hydroxyoctadecadienoic acid | 1.08 | 0.00152 | 1.02 |
| PE(17:0/0:0) | 1.11 | 0.00387 | 1.04 |
| (Z)-3-Hexenyl (e)-2-hexenoate | 1.31 | 0.00007 | 1.02 |
| Cinnamyl alcohol | 1.13 | 0.00377 | 1.01 |
| 2,3-Dimethyl-2-cyclohexen-1-one | 1.02 | 0.04505 | 1.01 |
| 2,2,6,7-Tetramethylbicyclo[4.3.0]nona-1(9),4-dien-8-one | 1.17 | 0.00175 | 1.02 |
| 3b-Allotetrahydrocortisol | 1.06 | 0.02509 | 1.74 |
| 9-oxo-11-(3-Pentyloxiran-2-yl)undec-10-enoate | 1.19 | 0.00178 | 1.02 |
| Dihydroxyeicosatrienoic acid | 1.17 | 0.00284 | 1.02 |
| Cortisol | 1.08 | 0.01723 | 1.11 |
| Ceanothenic acid | 1.16 | 0.01089 | 1.02 |
| Apo-10'-violaxanthal | 1.23 | 0.00302 | 1.03 |
| DiMe(3,5) | 1.31 | 0.00008 | 1.02 |
| LysoPC(14:0/0:0) | 1.16 | 0.01157 | 1.03 |
| 16-B1-phytoprostane | 1.26 | 0.00039 | 1.02 |
| Abscisic alcohol | 1.18 | 0.00152 | 1.02 |
| BHT-QM | 1.14 | 0.00217 | 1.01 |
| 5b-Cholestane-3a,7a,12a,23S,25-pentol | 1.1 | 0.00638 | 1.03 |
| Cepagenin | 1.12 | 0.0084 | 1.02 |
| N-lactoyl-phenylalanine | 1.01 | 0.00573 | 0.94 |
| Leukotriene B4 ethanolamide | 1.11 | 0.01472 | 1.02 |
| 10-Hydroxymatricaric acid | 1.04 | 0.03121 | 1.04 |
| 4-Isopropenyltoluene | 1.04 | 0.01207 | 1.04 |
| Cinncassiol E | 1.1 | 0.02257 | 1.02 |
| 3-(2-Methylpropanoyloxy)-8-(2-methylbutanoyloxy)-9,10-epoxy-p-mentha-1,3,5-triene | 1.13 | 0.00761 | 1.05 |
| 5-Methyldeoxycytidine | 1.01 | 0.00453 | 1.09 |
| H-D-Asp(OtBu)-OH | 1.08 | 0.00513 | 1.03 |
| Epicatechin 7-sulfate | 1.03 | 0.01332 | 0.85 |
| Homocitrulline | 1.06 | 0.02657 | 1.03 |
| Arginylproline | 1.03 | 0.01336 | 0.94 |
| Neuraminic acid | 1.06 | 0.02671 | 0.93 |
| (E)-2-Penten-1-ol | 1.21 | 0.00051 | 0.95 |

Abbreviations: FC, fold change, control mice/depressed mice; VIP, variable importance in projection.


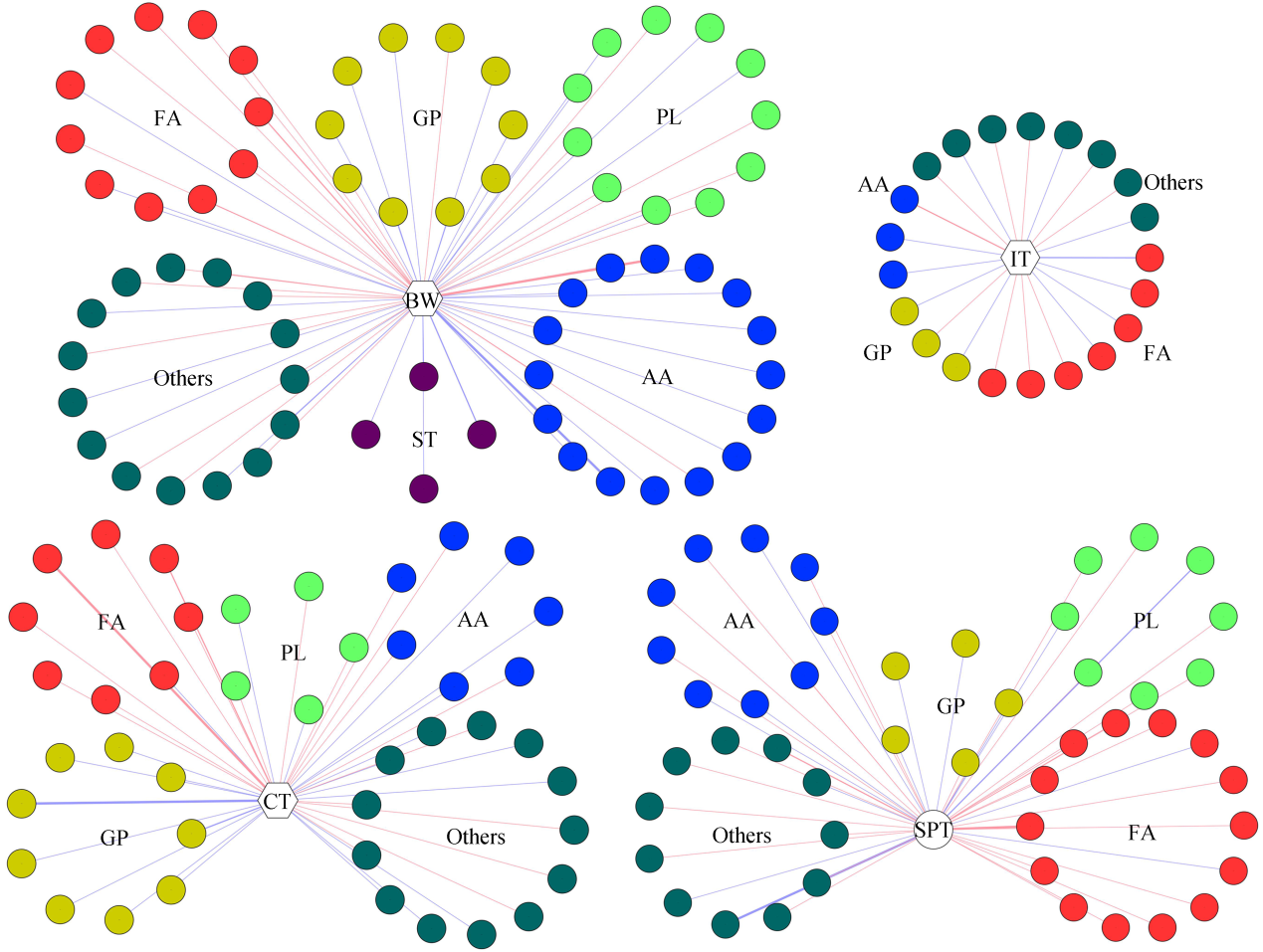


**Figure S1** **Correlations between differential microbial metabolites and depressive-like behaviors.** SPT, sucrose preference; IT, immobility time; CT, center time (%); BW, body weight; FA, fatty acyls; GP, glycerophospholipids; PL, prenol lipids; AA, amino acids; ST, steroids and steroid derivatives.

**Table S3 Correlations between differential bacterial taxa, differential metabolites and behaviors**

| **Variable 1** | **Variable 2** | **r** | **p** |
| --- | --- | --- | --- |
| 5-HTP | g_Muribaculum | -0.632 | 0.009 |
| 5-HTP | g_Intestinimonas | 0.506 | 0.046 |
| 5-HTP | Tryptophan | 0.629 | 0.009 |
| 5-HTP | SPT | 0.563 | 0.023 |
| Tryptophan | p_Firmicutes | 0.599 | 0.014 |
| Tryptophan | c_Bacilli | 0.75 | 0.001 |
| Tryptophan | g_Lactobacillus | 0.801 | 0.0002 |
| Tryptophan | o_Lactobacillales | 0.796 | 0.0002 |
| Tryptophan | g_Intestinimonas | 0.732 | 0.001 |
| Tryptophan | IT | -0.54 | 0.031 |
